# Rethinking Intellectual Disability from Neuro- to Astro-Pathology

**DOI:** 10.3390/ijms21239039

**Published:** 2020-11-27

**Authors:** Álvaro Fernández-Blanco, Mara Dierssen

**Affiliations:** 1 Centre for Genomic Regulation (CRG), The Barcelona Institute of Science and Technology, Dr. Aiguader 88, 08003 Barcelona, Spain; alvaro.fernandez@crg.eu; 2 Universitat Pompeu Fabra (UPF), Dr. Aiguader 88, 08003 Barcelona, Spain; 3 Centro de Investigación Biomédica en Red de Enfermedades Raras (CIBERER), 28029 Madrid, Spain

**Keywords:** astrocytes, neurodevelopmental disorders, memory deficits, Fragile X syndrome, Down syndrome

## Abstract

Neurodevelopmental disorders arise from genetic and/or from environmental factors and are characterized by different degrees of intellectual disability. The mechanisms that govern important processes sustaining learning and memory, which are severely affected in intellectual disability, have classically been thought to be exclusively under neuronal control. However, this vision has recently evolved into a more integrative conception in which astroglia, rather than just acting as metabolic supply and structural anchoring for neurons, interact at distinct levels modulating neuronal communication and possibly also cognitive processes. Recently, genetic tools have made it possible to specifically manipulate astrocyte activity unraveling novel functions that involve astrocytes in memory function in the healthy brain. However, astrocyte manipulation has also underscored potential mechanisms by which dysfunctional astrocytes could contribute to memory deficits in several neurodevelopmental disorders revealing new pathogenic mechanisms in intellectual disability. Here, we review the current knowledge about astrocyte dysfunction that might contribute to learning and memory impairment in neurodevelopmental disorders, with special focus on Fragile X syndrome and Down syndrome.

## 1. Introduction

Neurodevelopmental disorders are multifaceted conditions characterized by different degrees of intellectual disability and impairment in communication (verbal and non-verbal) and motor skills, among others. Intellectual disability is defined by an overall intelligence quotient below average and deficits in adaptive behaviors, with an early onset during childhood [1,2]. These disorders arise from genetic alterations and/or environmental factors that influence how the brain develops and have short and long-term consequences on cognition, social interaction, and behavior.

Traditionally, neurodevelopmental disorders such as Down syndrome (DS), Fragile X syndrome (FXS), or Rett syndrome have been considered as synaptopathies [3,4,5], characterized by neuronal alterations important for learning and memory, including abnormalities in dendritic architecture [6,7], with changes in the complexity of dendritic arborizations [7], and in spine number [6,7,8,9,10,11,12], shape and length [7,10,13], reflecting more immature spines [10,14]. These changes are accompanied by impaired synaptogenesis [15,16,17], alterations in synaptic transmission and broad deficits in synaptic plasticity [18,19,20,21], as revealed by mouse models for these syndromes.

Synaptic plasticity and, specifically, changes in the strength of synaptic connectivity of neurons that are activated at the time of learning are thought to be the basis of memory formation [22,23]. However, the alteration of the neuronal component does not completely explain the synaptic alterations and neurophysiological changes nor the behavioral and memory deficits observed in these neurodevelopmental disorders. This is possibly the reason why strategies that focused on restoring neuronal dendritic abnormalities, impaired synaptic plasticity, and different imbalances in neurotransmission only showed partial recoveries in the memory deficits associated with intellectual disability both in mouse models [9,24] and in humans [24,25,26]. Thus, we need to consider alternative mechanisms that may contribute to memory pathology.

Lately, the discovery of unique astroglial features that include roles in synaptic plasticity and memory function has broadened and refurbished the conception of brain function in health and disease. Recent reports underscoring the astrocyte capability to modulate neuronal circuit activity and to potentiate synapses [27,28] have led to consider that astrocytes are both necessary [29,30,31] and sufficient [28] for memory function. Moreover, studies on intellectual disabilities have recently uncovered potential contributions of astrocytes to their pathophysiology [16,32,33,34].

In fact, increasing body of evidence suggests that changes in astrocyte physiology and morphology might be involved in Alzheimer’s disease, FXS, or DS, among others [35,36,37,38,39]. In these pathological conditions, astrocytes modify their function and exhibit some common pathological features including an increase in the number and size of astrocytes together with increased expression of astroglial proteins such as S100 calcium-binding protein β (S100β) [40,41,42,43], a calcium binding protein coded by a HSA21 gene, and the glial fibrillary acidic protein GFAP [36,43,44,45], the main intermediate filament proteins of mature astrocytes. In fact, these pathophysiological changes in astrocytes have been linked with reduced neuronal activity [41], spine defects [14,32,34], and impaired memory performance [33,46,47,48].

In this review, we present the currently available data that state the involvement of astrocytes in synaptic transmission and in memory function both in the healthy and in the diseased brain. Given the emerging role of astrocytes in synaptic transmission, we propose specific mechanisms that may explain astrocyte dysfunction in neurodevelopmental disorders and can contribute, at least to some extent, to the memory deficits associated with these brain disorders. Specifically, we describe the main astroglial alterations in FXS and DS and propose new lines of research that may help to better understand the role of astrocytes in memory dysfunction.

## 2. Astrocyte Function in the Healthy Brain

Astrocytes are star-shaped cells whose function has been classically restricted to maintain brain homeostasis and to support neurons from a metabolic and structural perspective. Beyond these classical functions, astrocytes also play a role in synaptic physiology due to the expression of an extensive number of functional neurotransmitter receptors that allow them to sense different types of neurotransmitters [49,50]. Astrocytes are also able to release several neurotransmitters and neuromodulators, such as glutamate, GABA, ATP/adenosine and D-serine, to the synaptic cleft [51,52], through intracellular calcium concentration changes, so-called calcium oscillations [27,53,54]. This process, termed gliotransmission, has been extensively studied, as it influences synaptic physiology and potentiates or depresses synapses both in the short and long-term [27,28,55]. For this reason, it is widely accepted that astrocytes are an integral component of the synapse (tripartite synapse [56]) that, hand in hand with neurons, shape the synaptic transmission in the brain. However, the mechanisms by which astrocytes mediate these structural and functional responses have been a subject of intense debate [52,57,58].

Perisynaptic astrocytic processes (PAPs) containing neurotransmitter receptors embrace the synaptic cleft, isolating synapses in the tridimensional space. Taking into consideration that those fine astrocyte processes are covering around 60% of postsynaptic dendritic spines in the mature hippocampus [59], and that a single astrocyte can enwrap from 10,000 to 100,000 synapses [60], their potential to influence and shape synaptic transmission is gigantic. In addition, PAPs are remarkably dynamic, being as plastic as their neuronal counterparts due to their capability to change morphology, volume, and motility in response to specific neuronal activity patterns within minutes [61,62,63]. This astrocyte–synapse interplay is fundamental for neural transmission but also for other important processes related to cognitive function, such as spine maturation [62,64,65] and structural and functional synaptic plasticity by means of secreted molecules, such as thrombospondins [32,34,66], or by direct physical contact with synapses [62]. In fact, dendritic protrusions that are in contact with PAPs are typically more mature and stable than those present in the “bipartite synapses” [62].

## 3. Astrocyte Involvement in Learning and Memory in the Healthy Brain

The dynamic nature of astrocyte–synapse interactions is believed to sculpt and shape neuronal networks not only during neurodevelopment but also in adult stages. The prominent astroglial role in synaptic physiology suggests an astroglial involvement in memory function. Animal studies focused on functional neuron–astrocyte cross-talk have unraveled that the bidirectional communication between neurons and astrocytes is important for neuronal plasticity [28,31] and, more specifically, for memory function [28,29,30,31]. Recently, genetic tools such as chemogenetics and optogenetics have made it possible to specifically manipulate astrocytes in the brain, which may help understanding how astrocyte dysfunction may negatively impact memory function in neurodevelopmental disorders.

One of the first evidences supporting astrocytes involvement in memory processes came from transgenic mice overexpressing S100β [48]. Specifically, upregulation of S100β mRNA and protein levels in astrocytes led to impaired long-term potentiation (LTP) and exacerbated long-term depression (LTD) in hippocampal pyramidal neurons. These alterations in neuronal physiology were accompanied by a significant impairment in performance of the Morris water maze navigation test, a hippocampal-dependent task. Conversely, S100β-null mice showed enhanced LTP accompanied by enhanced spatial memory in the Morris water maze test and in contextual fear-conditioning [67]. Interestingly, exogenous application of S100β reversed the enhanced LTP of S100β-null mice, suggesting that astrocyte-secreted S100β can also influence neuronal activity. In fact, S100β has Ca^2+^ binding properties that reduce extracellular Ca^2+^ concentration and affect the neuronal firing pattern [68]. S100β also influences neuronal excitability since intracellular upregulation of S100β protein concentration increases astrocyte calcium oscillations and has been related with reduced neuronal excitability [41]. Indeed, the effects of S100β are concentration-dependent: while low concentrations have protective brain effects and promote the development and maturation of the central nervous system [69], high S100β concentrations are toxic, promote proinflammatory responses [70], and have detrimental consequences for neurons that include apoptotic cell death [71].

More recent studies that manipulated astroglial activity in a more mechanistic and time-constrained manner have demonstrated the astrocyte involvement in memory function [28,29,30,31]. For example, transgenic mice that allowed the selective expression of tetanus toxin (TeNT^Δ1^) in astrocytes resulted in the abolishment of the Ca^2+^ dependent neurotransmitter release and impaired, in vivo, the gamma oscillations in the hippocampus [30]. This fact has far-reaching implications since gamma rhythms are related with several cognitive processes such as attention, learning, and different types of memory [72]. Remarkably, the temporal blockade of astrocyte vesicular release by TeNT expression resulted in impaired recognition memory, while restoring the astrocyte capability to release gliotransmitters rescued these memory deficits. This study elegantly demonstrated in a very mechanistic manner that gliotransmission is necessary for particular types of memory and placed the astrocyte as a sophisticated and intricate player in memory function. However, it also left several open questions: How do astrocytes participate in learning? Can astrocytes play a role in the acquisition, consolidation, storage, and recall of memories?

Part of these questions were answered in a recent study in which astrocytes were selectively activated in the CA1 region of the hippocampus during memory acquisition, either by chemogenetics (GFAP-hM3Dq) or by optogenetics (GFAP-OptoGq) before (but not after) learning [28]. Astrocyte activation before learning resulted in memory enhancement of fear conditioning that was accompanied by an increase in the recruitment of active neurons during memory acquisition. This would suggest that more neurons support this particular memory in this particular brain region. Despite this study suggests that astrocyte activation might be important for memory acquisition, it does not prove whether astrocytes are activated in physiological conditions in the brain during memory operations.

Other studies specifically abolished astrocyte activity and/or signaling. For example, pharmacological inhibition of L-lactate production in astrocytes prevented long-term potentiation of CA3-CA1 synapses, in vivo, and impaired long-term episodic memory [31]. L-lactate is produced by astrocytes, and its transport from astrocytes to neurons is important for neuronal metabolism. This study suggests that astrocyte-neuron lactate transport is involved in long-term memory formation and for late-LTP. Another study that specifically activated the Gi pathway in astrocytes before learning, impaired remote (yet not the recent) memory recall [29]. G protein-coupled receptors play key roles in intercellular signaling in the brain [73]. While they inhibit neuronal activity, their effects are opposite in astrocytes, rising intracellular Ca^2+^ levels and promoting glutamate release [74]. Since recent-to-remote memory transition depends on the activation and recruitment of cortical regions such as the anterior cingulate cortex (ACC) [75,76,77], the authors activated the Gi pathway in CA1 astrocytes by GFAP-hM4Di designer receptor [29]. Interestingly, even though both CA1 and the ACC are important for recent and remote memories, Gi pathway activation in astrocytes by clozapine N-oxide, an hM4Di agonist, prevented neuronal activation (assessed by c-Fos expression) in the ACC but not in CA1. This suggests that astrocytes would modulate the functional connectivity between neurons in a projection-specific manner. As such, CA1 astrocytes would distinguish between distinct CA1 pyramidal neurons according to their projection target and would distinctively modulate their activity. It is noteworthy to mention that the manipulation (either activation [28] or inactivation [29]) of astrocyte activity with designer drugs itself, did not interfere with memory recall suggesting that astrocyte activity is necessary during memory acquisition but not for recent or remote memory recall.

In view of the recent discoveries involving astrocytes in memory function, one would consider that astrocyte dysfunction in any cognitive disorder might interfere with learning and memory. In the following section, we have selected the most relevant mechanisms by which astrocytes could impact and contribute to memory deficits in neurodevelopmental disorders, focusing on FXS and DS.

## 4. Astrocyte Dysfunction in Neurodevelopmental Disorders

The involvement of astroglia in the pathophysiology of several cognitive disorders is supported by evidence that report that numerous glial genes are misregulated in neurodevelopmental disorders [40,78]. The proteins encoded by these genes play important roles in the brain, including their involvement in cell cycle progression, neuronal differentiation, and repairing the neuronal damage. Together with these genetic alterations, astrocytes also display aberrant morphology [42,79] and physiology [41] that have been directly shown to contribute not only to synaptic defects and changes in neuronal excitability [41] but also to neuronal survival [80,81].

It is frequent to detect different degrees of astrocyte reactivity (or astrogliosis) in many brain disorders [36,42,79]. The term astrogliosis refers to changes at the molecular, cellular, and functional level that appear as a response to brain damage or in genetic brain disorders [82]. The changes in astroglial function vary depending on the severity of the lesion or the genetic alteration and have repercussions on adjacent neurons. In general, astrogliosis involves morphological and physiological alterations, such as an increase in the number and size of the astrocytes, and changes in the expression of astroglial proteins (GFAP and S100β) [82]. Astrogliosis promotes, together with peripheral macrophages and microglia, an adaptive state that helps facing the origin of the brain insult (infection, hemorrhage, genetic disturbance, etc.) by phagocytizing external factors, eliminating toxic neuronal debris, and/or promoting neuronal survival [82,83,84]. However, in cognitive disorders such as DS, FXS, or Alzheimer’s disease, astrocytes are in a chronic “reactive state” (or abnormal astrogliosis), a continuous dysfunctional mode that can be maladaptive and contribute to the progression of neurodegeneration of these brain disorders [42,85].

Given the role of astrocytes in the regulation of synaptic function, it is not surprising that changes in astrocyte activity, protein secretion, deregulation in gene expression, or modification in the astroglial membrane channel composition that occur in neurodevelopmental disorders might impair synaptic transmission and, therefore, memory function. In the next section, we review some of the most relevant studies demonstrating that astrocytes are involved in the synaptic pathology in DS and FXS, the two most common genetic forms of intellectual disability.

### 4.1. Astrocyte Pathology in Fragile X Syndrome (FXS)

FXS is a genetic condition caused by an expansion of the CGG triplet within the fragile X mental retardation 1 gene (*FMR1)* that leads to its transcriptional silencing. *FMR1* is located in the X chromosome and encodes for the mRNA binding protein Fragile X Mental Retardation Protein 1 (FMRP) that regulates protein synthesis. The absence of FMRP interferes with brain development and contributes to FXS pathophysiology [86,87]. Several lines of evidence suggest that astroglia might also contribute to synaptic function impairment and memory deficits of individuals with FXS [16,32,33]. However, studies on astrocyte pathology in FXS are sparse and inconsistent: one group reports no astrogliosis seen in post-mortem brains of persons with FXS [88] while other describes a gliosis in the CA4 hippocampal region of two postmortem FXS brains [89].

**Figure 1:**
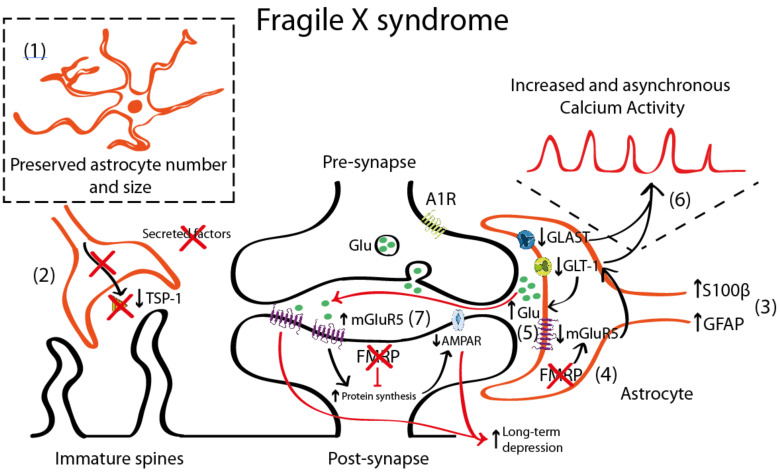
Schematic representation illustrating the astrocyte-synapse alterations in Fragile X syndrome (FXS). (**1**) Astrocyte number is preserved. (**2**) Reduced astrocyte secreted thrombospondin (TSP-1) prevents spine maturation resulting in more abundant filopodia (immature) spines. (**3**) Increased S100 calcium-binding protein β (S100β) and GFAP expression has been described in astrocytes. However, this altered expression has not been directly linked with their activity or function. (**4**) Fragile X Mental Retardation Protein (FMRP) absence in FXS astrocytes leads to metabotropic glutamate receptor 5 (mGluR5) downregulation in astrocytes (yet not in neurons) that negatively regulates glutamate transporter 1 (GLT-1) expression. Impaired glutamate transport due to decreased astrocyte glutamate-aspartate transporter 1 (GLAST-1) and GLT-1 expression increases extracellular glutamate levels (**5**) and astroglial calcium oscillations (**6**). This excess of glutamate might activate the postsynaptic mGluR5, which is overexpressed in neurons. (**7**) mGluR5 and FMRP oppositely regulate mRNA translation at the synapse: mGluR5 promotes it and FMRP prevents it. Therefore, increased mGluR5 expression and lack of FMRP in FXS leads to a disbalance in protein expression levels that account for many of the syndromic features of FXS including an α-amino-3-hydroxy-5-methyl-4-isoxazolepropionic acid receptor (AMPAR) internalization that leads to an exaggerated mGluR5-mediated long-term depression (LTD) that is reported in *Fmr1* knock-out (KO) mice.

One of the most commonly used mouse models for the study of FXS is the *Fmr1* knock-out (KO) that recapitulates most of the neuronal alterations and the phenotypical traits of FXS [90]. This model lacks the expression of FMRP protein in neurons and astrocytes being thus an interesting tool to uncover the role of astrocyte in FXS. It is still unclear, however, whether FMRP protein has similar or different functions when expressed in neurons or in astrocytes. In FXS, the FMRP protein regulates metabotropic glutamate receptor 5 (mGluR5) expression in astrocytes, but not in neurons. In *Fmr1* KO astrocytes, the absence of FMRP leads to a downregulation of mGluR5 that leads to reduced expression of glutamate transporter 1 (GLT-1) and, subsequently, to decreased glutamate uptake in astrocytes [91] (Figure 1). Increased mGluR5 signaling has been long proposed to account for the syndromic features and the cognitive deficits in FXS [92,93]. In fact, the acute and chronic pharmacological inhibition of mGluR5 in adult *Fmr1* KO mice restores dendritic alterations including aberrant dendritic morphology, increases protein synthesis, and rescues memory deficits associated with FXS [94,95]. As mentioned, mGluR5 is upregulated in neurons [96] but downregulated in astrocytes [91]. Thus, the contribution of mGluR5 dysregulation to FXS pathophysiology is more complex than expected. mGluR5 activation has been associated with a form of synaptic depression, called mGluR5-mediated LTD resulting from the internalization of surface-expressed α-amino-3-hydroxy-5-methyl-4-isoxazolepropionic acid receptor (AMPAR) as a response to mGluR5 activation in neurons [97,98]. This is accompanied by reduced presynaptic release of glutamate [99] that, overall, leads to an exaggerated LTD in *Fmr1* KO mice [100]. Conversely, mGluR5 downregulation in *Fmr1* KO astrocytes has been associated with lower astroglial GLT-1 levels [91]. This might contribute to reduced glutamate reuptake and, therefore, to increased extracellular glutamate levels which subsequently might activate mGluR5 receptors in postsynaptic neurons, thus promoting mGluR5-mediated LTD.

Interestingly, increased GFAP expression, a marker of astrocyte activation, has been reported in the cortex, hippocampus, and striatum of *Fmr1* KO mice [101], suggesting a reactive gliosis in these particular brain regions. In general, increased GFAP expression is accompanied by an increase in the number of astrocytes [82]. Nevertheless, there is only one description of persistent astrogliosis (increased GFAP and S100β expression) in the cerebellum of *Fmr1* KO mice [102], but no systematic quantification of the astroglial number has been performed in FXS. Complementary to these data, astrocyte physiology was also reported to be altered in a mouse model with a gain-of-function of the premutation CGG (preCGG) repeat within the *FMR1* gene. preCGG knock-in mice showed increased and asynchronous calcium activity that was explained by increased glutamate levels in the extracellular space due to reduced expression of glutamate transporters such as the glutamate-aspartate transporter 1 (GLAST-1) and GLT-1 in astrocytes [103].

In cellular FXS models, control hippocampal neurons grown in co-culture with FXS astrocytes, showed aberrant dendritic morphology and decreased expression of synaptic markers (PSD-95) [103]. Conversely, when co-cultured with control astrocytes, hippocampal FXS neurons developed normally. These dendritic alterations were explained by a reduction in the expression of thrombospondin (TSP-1) in *Fmr1* KO mice astrocytes [32]. TSP-1 is synthesized and secreted by astrocytes [104], promoting synaptogenesis [66] and neurite outgrowth [105] both during the neurodevelopment [106] and in the adult brain [107]. Interestingly, both culturing *Fmr1* KO hippocampal neurons with astrocyte-conditioned media of FMRP-expressing (control) astrocytes and the exogenous application of TSP-1, prevented dendritic spine defects in *Fmr1* KO neurons. This indicates that astrocyte-secreted TSP-1 is a potent modulator of dendritic morphology and that reduced TSP-1 in FXS astrocytes would contribute to dendritic alterations in FXS neurons.

In agreement with these results, an astrocyte-specific *Fmr1* KO mouse model shows similar dendritic and cognitive alterations than *Fmr1* KO mice [33]. However, restoring FMRP expression specifically in *Fmr1* KO astrocytes was not sufficient to restore dendritic and learning deficits associated with FXS suggesting that astrocyte dysfunction does not completely account for FXS pathophysiology.

### 4.2. Astrocyte Pathology in Down Syndrome

DS is the most prevalent cause of intellectual disability of genetic origin. It is due to the presence of a third copy of HSA21, which results in deregulated gene expression leading to altered brain function. DS brain manifestations include changes in the volume and connectivity of certain brain regions such as the cerebral cortex, cerebellum, and hippocampus [108,109], and neuroarchitectural alterations such as spine dysgenesis [14,110], decreased spine density [8,9], and dendritic atrophy [7]. These alterations may deeply perturb information processing in structures related to cognitive functions such as hippocampus and cortex [110] and are assumed to underlie some cognitive impairments in DS, ranging from learning difficulties to spatial memory deficits [111].

**Figure 2:**
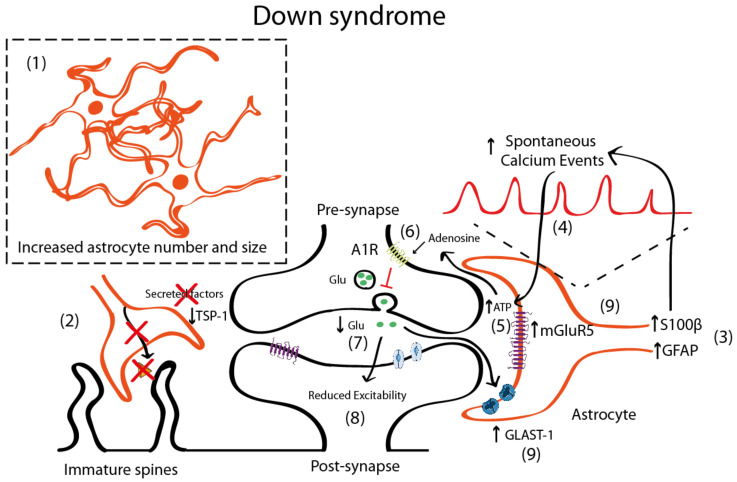
Schematic representation illustrating the astrocyte-synapse alterations in DS. (**1**) Astrocyte number and volume is increased in DS. (**2**) Reduced astrocyte secreted TSP-1 prevents spine maturation resulting in more frequent filopodia (immature) spines. (**3**) Increased S100β and GFAP expression has been described in astrocytes. S100β upregulation has been linked with increased astrocyte calcium oscillations (**4**). Increased astrocyte activity leads to adenosine triphosphate (ATP) release to the synaptic cleft (**5**) that is hydrolyzed to adenosine and activates A1 adenosine receptors (A1R) (**6**). A_1_R activation prevents glutamate release from the presynaptic terminal and, consequently (**7**) depresses synaptic transmission (**8**). Even though mGluR5 is upregulated in astrocytes (and probably in neurons), no mechanistic studies have been performed to uncover the contribution of mGluR5 to DS pathophysiology. (**9**) Reduced glutamate concentrations can be contributed by increased expression of the glutamate transporter GLAST-1 that leads to increased astroglial glutamate uptake.

In DS, there is an increased number of astrocytes [42,85,112] (Figure 2). In post-mortem brains of individuals with DS, astrocytes are more abundant, bigger, and express more astroglial markers (S100β, GFAP) than age-matched controls [42,112]. Similar observations were reported in Ts65Dn, a partial trisomic mouse model for DS [113]. Nevertheless, there are some discrepancies that suggest that GFAP is reduced in particular brain areas [114,115]. DS fetuses have a higher percentage of cells with astrocytic phenotype in the hippocampus [116], thus indicating a shift from neurogenesis to gliogenesis. This neurogenic-to-gliogenic switch was confirmed in induced pluripotent stem cells (iPSCs) from monozygotic twins discordant for trisomy 21 in which a shift towards the astroglial phenotype was detected in the transcriptional signature of DS-iPSC-derived cells, as shown by the increased expression of *GFAP*, *S100β*, and *Vimentin* [117]. HSA21 genes, such as the Dual specificity tyrosine-phosphorylation-regulated kinase 1A (*DYRK1A)*, may play a role through the activation of the astrogliogenic transcription factor signal transducer and activator of transcription (STAT) that subsequently induces precocious astrogliogenesis by switching the neural progenitor fate towards the astroglial phenotype [118]. These findings suggest that gene expression deregulation in DS, and specifically changes in the DYRK1A-STAT signaling pathway, control the onset of the gliogenic switch and favor the neural progenitor cell fate towards astrogliogenesis.

The increased astrocyte number [42,85,112] is accompanied by a reduction in the neuronal population [116,119], due to impaired proliferation [116,120] and increased apoptosis [116,121]. This implies that the neuron to glia ratio would be reduced in DS, which could have profound implications. In fact, astrocytes are distributed in non-overlapping synaptic territories in the tridimensional space, which allows to modulate the synaptic transmission of several neurons at a time. These separated anatomical domains, called synaptic islands [122], are particularly relevant, as they might prevent the redundancy of different astrocytes controlling the same or neighboring synapses. In the DS scenario, astrocytes are increased both in number and in volume while neuronal numbers are reduced. These physical changes may not only affect how neurons and astrocytes are distributed and positioned in the tridimensional space but would also determine how many neurons a single astrocyte contacts. As a consequence, the astrocyte-associated area of influence would be affected, with probable consequences on neuronal communication. We speculate that the changes in the tridimensional arrangement could lead to a synaptic island overlap, promoting a misregulation and redundancy in the astroglial control of synaptic transmission.

In DS, astrocytosis is maintained throughout life and is accompanied by an immature astroglial phenotype with decreased interlaminar processes [123]. Moreover, astroglial physiology is altered, as shown by the increased spontaneous calcium oscillations in DS astrocytes [41]. This may impact neuronal function, since intracellular calcium transients in astrocytes induce the release of gliotransmitters. In fact, DS-iPSCs-derived astrocytes exhibited increased calcium activity, which was shown to subsequently reduce the excitability of co-cultured neurons [41]. The increase of calcium oscillations was attributed to S100β overexpression, since normalization of S100β expression restored calcium activity to control levels [41]. Interestingly, the reduction of evoked field potentials in DS iPSCs co-cultured neurons was prevented by blocking the A_1_ adenosine receptor (A_1_R) with 8-Cyclopentyl-1,3-dipropyl xanthine (DPCPX, a potent A_1_R antagonist). This would suggest that either A_1_R is overexpressed in neurons—a fact that to the best of our knowledge has not yet been described. A second possible explanation would be that increased adenosine concentration driven by the hydrolysis of an excess of ATP released consequent to the astrocyte hyperactivity would activate neuronal A_1_Rs. Adenosine has been shown to activate A_1_R in neighboring synapses [124,125] and A_1_R activation inhibits glutamate release [126,127], consistent with the reduced glutamate levels in DS that lead to an excitatory/inhibitory imbalance [128,129,130]. Specifically, the levels of glutamate are reduced in several brain regions including the parahippocampal gyrus [128] in the hippocampus [129] and in peripheral tissues [130], which could produce an overall reduction in the neuronal activity. These reduced glutamate levels could also be explained by the increased expression of the glutamate transporter GLAST-1 in DS astrocytes leading to a higher glutamate uptake compared to control astroglia [131]. Conversely, the levels of GLT-1 were preserved in DS astrocytes.

Although there is still some controversy, it seems that in DS, there is also an increased inhibition [132,133,134], since blocking GABA_A_ receptors restores LTP deficits in Ts65Dn mice [135], while excitation is preserved [132] or slightly reduced [136]. Some studies suggested that the number of excitatory synapses would be reduced [136], while inhibitory synapses would be preserved [136]. In DS, there is an upregulation of mGluR5 both in fetal and adult DS brains [137] and this mGluR5 upregulation is astrocyte-specific [137]. However, no mechanistic studies associating mGluR5 with synaptic alterations either at the structural or functional level have been performed in mouse models for DS. Nevertheless, the deregulation of mGluR5 signaling in neurons and/or astrocytes can ultimately lead to alterations in the astrocyte–synapse cross-talk that is essential for synaptic transmission and contribute, at least to some extent, to the memory deficits associated with DS.

Interestingly, and similar to the FXS scenario, the co-culture of rat hippocampal neurons that were grown on top of human DS astrocytes showed reduced levels of TSP-1 (around 60% lower compared to WT astrocytes) which led to a reduction in the neuron spine number and more immature (filopodia) spines compared to neurons cultured with control astrocytes [14,34]. Conversely, the addition of TSP-1 in co-cultures of neurons and DS astrocytes restored the alterations in the dendritic spines, suggesting that TSP-1 dysfunction in DS contributes to aberrant dendritic morphology.

Studies focusing on astrocyte pathology on DS are scarce and limited and very few have explored in detail the mechanisms by which astrocytes could contribute to synaptic alterations in DS. Thus, exploring the mechanisms that might lead to astrocyte–synapse communication such as mGluR5 signaling, purinergic transmission, or deficits in TSP-1 secretion can provide new levels of understanding about the contribution of astrocyte dysfunction to memory deficits in DS.

## 5. Astrocytic Phenotypes in DS and FXS: Same Players for Different Phenotypes?

Neurodevelopmental disorders are among the most complex medical conditions, in terms of pathophysiological mechanisms and possible treatments. Most of them lead to intellectual disability, including both cognitive and behavioral impairments that have been traditionally ascribed to neuronal defects. In the last 20 years, astrocytes have emerged as key players in neurotransmission, helping to address longstanding questions in the intellectual disability field with an entirely novel perspective. Astrocytes also hold great promises for cognitive and behavioral repair.

Given that FXS and DS are the most preeminent neurodevelopmental disorders associated with intellectual disability, we have centered our review in those disorders, and on the astrocyte alterations that may impair fundamental mechanisms required for memory function including neuronal communication and synaptic function. Interestingly, we found several studies in FXS and in DS indicating that these disorders share deficits in astrocyte structure, gene expression, and/or function. How those may contribute to the different intellectual disability profiles in FXS and DS is still unexplored, as are also other mechanisms by which astrocytes could contribute to memory deficits.

Interestingly, while there is a lifelong astrocytosis in DS [85], this phenotype is not detected in FXS. One of the most important but also mysterious features of the astrocyte–neuron communication is the existence of so-called synaptic islands. Between 50% and 60% of hippocampal synapses are engulfed by extensive astrocytic ensheathment that prevents spillover and spatially isolates individual synapses from each other and from the extra-synaptic space [59,122]. However, the study of these non-overlapping astroglial domains have not been explored neither in FXS nor in DS. This is particularly relevant because it can provide new levels of understanding about how neurons and astrocytes interact in the tridimensional space and how astrocyte–synapse cross-talk varies over time. In the DS scenario, the decreased neuron to astrocyte ratio would imply that the astroglial domains would be disturbed. The increased number and volume of astrocytes would lead either to smaller astroglial domains or to overlapping synaptic islands and, therefore, the boundaries between astroglial domains would be more diffuse.

However, due to the dynamic nature of astrocytes, which are remarkably motile and can engage and disengage from synapses spontaneously or in response to physiological (or pathological) stimuli [61,62,63], these synaptic islands are expected to change over time. Thus, even though no substantial changes in astrocyte number or volume have been described in FXS that might indicate changes in the neuron to astrocyte ratio, it could happen that the dynamics of the extensive astrocytes processes in FXS might vary, thus changing the defined astroglial locations in which astrocytes modulate a specific number of synapses. Likewise, this hypothetical shift in the motility of the astroglial domains might also produce that the well-established boundaries between synaptic islands become more diffuse, as in DS, leading to dysregulation of synaptic transmission in FXS.

In both DS and FXS, even though no direct conclusions can be extracted, one would expect that synaptic transmission would be affected somehow, probably by adding some redundancy in the system since more astrocytes would be in charge of controlling a similar number of synapses or because the different astroglial topology would modify the synaptic astroglial coverage that is important for synaptic transmission.

Furthermore, the scope of the astroglial contribution to synaptic alterations in neurodevelopmental disorders also includes other important players such as proteins related with the neurotransmitter and ionic homeostasis such as glutamate transporters (GLT-1 and GLAST-1), glutamine synthetase, aquaporins, potassium channels, and lactate transporters. How specific alterations in these proteins might contribute to memory deficits in FXS and DS might include changes in neuron excitability due to impaired astrocyte–synapse cross-talk. The study of these alterations can provide invaluable clues to comprehend the molecular basis of intellectual disability in these brain disorders. For example, differences in glutamate transporters such as GLT-1 and GLAST-1 modifies the glutamate levels [91] that influence neuronal activity. In FXS, reduced GLT-1 and GLAST-1 increase glutamate levels [91,103] lead possibly to a depression of the synaptic transmission by mGluR5-mediated LTD, or are causative, at least to some extent, for the hyperexcitability that has been widely described in FXS [138,139,140]. Conversely, in DS, GLAST-1 is overexpressed in astrocytes and leads to an increased astroglial glutamate uptake [131], which reduces the available glutamate in the extracellular space and probably reduces neuronal activity. As such, changes in the expression of several receptors such as mGluR5, which are downregulated in FXS astrocytes [91] and upregulated both in FXS neurons [96] and in DS astrocytes [141] can affect how neurons and astrocytes interact at the synaptic level. In neurons, mGluR5 is functionally coupled to N-methyl-D-aspartic acid receptors (NMDAR) by means of a protein scaffold constituted by Homer isoforms and Shank, among others [142,143]. In fact, mGluR5 activation has been shown to enhance NMDAR function [144,145] but also to promote mGluR5-dependent LTD [146], and these differences are probably due to different patterns of incoming glutamatergic signals in the postsynaptic neuron. Despite the well-known roles of mGluR5 in synaptic plasticity in neurons [147,148], very few studies have studied the contribution of astroglial mGluR5 signaling to the synaptic transmission either in FXS or in DS. Unraveling differential roles of mGluR5 in neurons and astrocytes in FXS would help to better dissect and elucidate the potential mechanistic interactions between neurons and astrocytes associated with mGluR5 dysfunction. Correspondingly, in DS, the investigation of mGluR5 function both in neurons and astrocytes would help to better understand the dysfunctional cross-talk in the tripartite synapse. In DS, mGluR5 is upregulated in astrocytes [141] (and probably in neurons [137]), yet no mechanistic studies have demonstrated the potential implications of mGluR5 dysfunction, neither in neurons nor in astrocytes.

Other important contributors to the intellectual disability phenotype would involve impairments in spine maturation and synaptogenesis not only during neurodevelopment but also in adult stages, as seen by the TSP-1 downregulation also in FXS and in DS [14,32,34]. Taking into consideration that the formation of new synapses and the strengthening of the existing ones is indispensable for memory function [22,23], these astrocyte-driven dendritic deficits that hamper synaptic function might have profound implications for the establishment of specific connectivity maps that support memories over time both in FXS and DS. Thus, strategies targeting TSP-1 deficits both in FXS and in DS can provide new lines of investigation to tackle dendritic alterations that are believed to contribute to memory deficits associated with these brain disorders.

Another interesting approach that could shed light into the astroglial contribution to memory deficits could be the restoration of epigenetic mechanisms that deregulate gene expression. For instance, in FXS hypermethylation of CGG triplets avoid FMRP expression in neurons [149,150]. In DS, there is a hypermethylation that represses gene expression in certain chromosomes [151,152,153] together with a hypoacetylation described in Ts65Dn hippocampus [154], a mouse model of DS, that would promote, in general, an downregulation of several memory-related genes. Recently, it has been possible to specifically edit the epigenetic footprints in a gene-specific manner. In fact, the demethylation of CGG repeats in iPSCs-derived FXS neurons induces an active chromatin state and restores most of the dendritic alterations to WT levels [150]. However, this approach has not been directed yet to restore genetic alterations due to epigenetic imbalance in the astroglial population. For instance, it could be interesting to restore GFAP and/or S100β expression in DS in which these genes are upregulated in order to see potential recoveries at the synaptic level and, possibly, also in cognition.

## 6. Astrocyte Involvement in Memory Pathology in Neurodevelopmental Disorders: A Look into the Future

In conclusion, our review shows that, to date, only few studies have specifically targeted the astrocytes involved in the cognitive and behavioral deficits associated with intellectual disability. Nevertheless, all the alterations detected in FXS and DS at the synaptic level in which astrocytes are involved, suggest that their perturbation has a detrimental effect on these disorders, and we propose specific models that would explain these alterations in FXS and in DS.

This is an exciting time for astrocyte research since we are beginning to understand unique and fascinating astroglial features that go beyond the outdated “neurocentric” vision. Shortly, and every time more frequently, we will hear about new discoveries that involve the astrocytes in different brain functions such as attention, sleep, executive functions, social behavior, and in several types of memory since we are now able to manipulate the astrocyte activity while mice undergo different tasks in a very time-constrained manner. However, more importantly, we may envisage that astrocytes could become a promising target for new treatments for a number of brain disorders.

## Abbreviations

DS	Down syndrome
FXS	Fragile X syndrome
S100β	S100 calcium-binding protein β
HSA21	Homo Sapiens Autosome 21
GFAP	Glial Fibrillary Acidic Protein
GABA	Gamma aminobutyric acid
ATP	Adenosine triphosphate
PAP	Perisynaptic astrocytic process
LTP	Long-term potentiation
LTD	Long-term depression
TeNT	Tetanus Neurotoxin
hM3Dq	human Gq-coupled M3 muscarinic receptor
CA1	Cornu Ammonis 1
CA3	Cornu Ammonis 3
ACC	Anterior Cingulate Cortex
*FMR1*	fragile X mental retardation 1
FMRP	Fragile X Mental Retardation Protein
CA4	Cornu Ammonis 4
KO	Knock-out
mGluR5	Metabotropic Glutamate Receptor 5
GLT-1	Glutamate Transporter 1
GLAST-1	Glutamate Aspartate Transporter 1
TSP-1	Thrombospondin-1
iPSC	Induced pluripotent stem cells
YRK1A	Dual-specificity tyrosine phosphorylation-regulated kinase-1
STAT	Signal Transducer and Activator of Transcription
NPC	Neural Progenitor Cell
A1R	Adenosine 1 Receptor
DPCPX	8-Cyclopentyl-1,3-dipropyl xanthine

## References

[1] Dierssen M. (2020). Top ten discoveries of the year: Neurodevelopmental disorders. Free Neuropathol..

[2] Papazoglou A., Jacobson L.A., McCabe M., Kaufmann W., Zabel T.A. (2014). To ID or Not to ID? Changes in Classification Rates of Intellectual Disability Using DSM-5. Intellect. Dev. Disabil..

[3] Ardiles A.O., Grabrucker A.M., Scholl F.G., Rudenko G., Borsello T. (2017). Molecular and Cellular Mechanisms of Synaptopathies. Neural Plast..

[4] Luo J., Norris R., Gordon S., Nithianantharajah J. (2018). Neurodevelopmental synaptopathies: Insights from behaviour in rodent models of synapse gene mutations. Prog. Neuro Psychopharmacol. Biol. Psychiatry.

[5] Dierssen M., Ramakers G.J.A. (2006). Dendritic pathology in mental retardation: From molecular genetics to neurobiology. Genes Brain Behav..

[6] Irwin S.A., Galvez R., Greenough W.T. (2000). Dendritic Spine Structural Anomalies in Fragile-X Mental Retardation Syndrome. Cereb. Cortex.

[7] Dierssen M., Benavides-Piccione R., Martínez-Cué C., Estivill X., Flórez J., Elston G., DeFelipe J. (2003). Alterations of neocortical pyramidal cell phenotype in the Ts65Dn mouse model of Down syndrome: Effects of environmental enrichment. Cereb. Cortex.

[8] Belichenko P.V., Masliah E., Kleschevnikov A.M., Villar A.J., Epstein C.J., Salehi A., Mobley W.C. (2004). Synaptic structural abnormalities in the Ts65Dn mouse model of down syndrome. J. Comp. Neurol..

[9] Catuara-Solarz S., Espinosa-Carrasco J., Erb I., Langohr K., Gonzalez J.R., Notredame C., Dierssen M. (2016). Combined Treatment with Environmental Enrichment and (-)-Epigallocatechin-3-Gallate Ameliorates Learning Deficits and Hippocampal Alterations in a Mouse Model of Down Syndrome. eNeuro.

[10] Comery T.A., Harris J.B., Willems P.J., Oostra B.A., Irwin S.A., Weiler I.J., Greenough W.T. (1997). Abnormal dendritic spines in fragile X knockout mice: Maturation and pruning deficits. Proc. Natl. Acad. Sci. USA.

[11] Hinton V.J., Brown W.T., Wisniewski K., Rudelli R.D. (1991). Analysis of neocortex in three males with the fragile X syndrome. Am. J. Med. Genet..

[12] Xu X., Miller E.C., Pozzo-Miller L. (2014). Dendritic spine dysgenesis in Rett syndrome. Front. Neuroanat..

[13] Landi S., Putignano E., Boggio E.M., Giustetto M., Pizzorusso T., Ratto G.M. (2011). The short-time structural plasticity of dendritic spines is altered in a model of Rett syndrome. Sci. Rep..

[14] Garcia O., Torres M., Helguera P., Coskun P., Busciglio J. (2010). A Role for Thrombospondin-1 Deficits in Astrocyte-Mediated Spine and Synaptic Pathology in Down’s Syndrome. PLoS ONE.

[15] Stagni F., Salvalai M.E., Giacomini A., Emili M., Uguagliati B., Xia E., Grilli M., Bartesaghi R., Bartesaghi R. (2019). Neonatal treatment with cyclosporine A restores neurogenesis and spinogenesis in the Ts65Dn model of Down syndrome. Neurobiol. Dis..

[16] Jacobs S., Doering L.C. (2010). Astrocytes Prevent Abnormal Neuronal Development in the Fragile X Mouse. J. Neurosci..

[17] Fukuda T., Itoh M., Ichikawa T., Washiyama K., Goto Y.-I. (2005). Delayed Maturation of Neuronal Architecture and Synaptogenesis in Cerebral Cortex ofMecp2-Deficient Mice. J. Neuropathol. Exp. Neurol..

[18] Kleschevnikov A.M., Belichenko P.V., Villar A.J., Epstein C.J., Malenka R.C., Mobley W.C. (2004). Hippocampal Long-Term Potentiation Suppressed by Increased Inhibition in the Ts65Dn Mouse, a Genetic Model of Down Syndrome. J. Neurosci..

[19] Zhao M.-G., Toyoda H., Ko S.W., Ding H.-K., Wu L.-J., Zhuo M. (2005). Deficits in Trace Fear Memory and Long-Term Potentiation in a Mouse Model for Fragile X Syndrome. J. Neurosci..

[20] Martin H.G.S., Lassalle O., Brown J.T., Manzoni O.J. (2015). Age-Dependent Long-Term Potentiation Deficits in the Prefrontal Cortex of theFmr1Knockout Mouse Model of Fragile X Syndrome. Cereb. Cortex.

[21] Weng S.-M., McLeod F., Bailey M.E.S., Cobb S.R. (2011). Synaptic plasticity deficits in an experimental model of rett syndrome: Long-term potentiation saturation and its pharmacological reversal. Neuroscience.

[22] Fred Attneave M.B., Hebb D.O. (1950). The Organization of Behavior; A Neuropsychological Theory. Am. J. Psychol..

[23] Liu X., Ramirez S., Pang P.T., Puryear C.B., Govindarajan A., Deisseroth K., Tonegawa S. (2012). Optogenetic stimulation of a hippocampal engram activates fear memory recall. Nat. Cell Biol..

[24] De La Torre R., De Sola S., Pons M., Duchon A., De Lagran M.M., Farré M., Fitó M., Benejam B., Langohr K., Rodriguez J. (2014). Epigallocatechin-3-gallate, a DYRK1A inhibitor, rescues cognitive deficits in Down syndrome mouse models and in humans. Mol. Nutr. Food Res..

[25] De La Torre R., De Sola S., Hernandez G., Farré M., Pujol J., Rodriguez J., Espadaler J.M., Langohr K., Cuenca-Royo A., Principe A. (2016). Safety and efficacy of cognitive training plus epigallocatechin-3-gallate in young adults with Down’s syndrome (TESDAD): A double-blind, randomised, placebo-controlled, phase 2 trial. Lancet Neurol..

[26] Jacquemont S., Berry-Kravis E., Hagerman R., Von Raison F., Gasparini F., Apostol G., Ufer M., Portes V.D., Gomez-Mancilla B. (2014). The challenges of clinical trials in fragile X syndrome. Psychopharmacology.

[27] Perea G., Araque A. (2007). Astrocytes Potentiate Transmitter Release at Single Hippocampal Synapses. Science.

[28] Adamsky A., Kol A., Kreisel T., Doron A., Ozeri-Engelhard N., Melcer T., Refaeli R., Horn H., Regev L., Groysman M. (2018). Astrocytic Activation Generates De Novo Neuronal Potentiation and Memory Enhancement. Cell.

[29] Kol A., Adamsky A., Groysman M., Kreisel T., London M., Goshen I. (2020). Astrocytes contribute to remote memory formation by modulating hippocampal–cortical communication during learning. Nat. Neurosci..

[30] Lee H.S., Ghetti A., Pinto-Duarte A., Wang X., Dziewczapolski G., Galimi F., Huitron-Resendiz S., Piña-Crespo J.C., Roberts A.J., Verma I.M. (2014). Astrocytes contribute to gamma oscillations and recognition memory. Proc. Natl. Acad. Sci. USA.

[31] Suzuki A., Stern S.A., Bozdagi O., Huntley G.W., Walker R.H., Magistretti P.J., Alberini C.M. (2011). Astrocyte-Neuron Lactate Transport Is Required for Long-Term Memory Formation. Cell.

[32] Cheng C., Lau S.K.M., Doering L.C. (2016). Astrocyte-secreted thrombospondin-1 modulates synapse and spine defects in the fragile X mouse model. Mol. Brain.

[33] Hodges J.L., Yu X., Gilmore A., Bennett H., Tjia M., Perna J.F., Chen C.-C., Li X., Lu J., Zuo Y. (2017). Astrocytic Contributions to Synaptic and Learning Abnormalities in a Mouse Model of Fragile X Syndrome. Biol. Psychiatry.

[34] Torres M.D., Garcia O., Tang C., Busciglio J. (2017). Dendritic spine pathology and thrombospondin-1 deficits in Down syndrome. Free. Radic. Biol. Med..

[35] Dossi E., Vasile F., Rouach N. (2018). Human astrocytes in the diseased brain. Brain Res. Bull..

[36] Simpson J., Ince P., Lace G., Forster G., Shaw P., Matthews F., Savva G., Brayne C., Wharton S.B. (2010). Astrocyte phenotype in relation to Alzheimer-type pathology in the ageing brain. Neurobiol. Aging.

[37] Kuchibhotla K.V., Lattarulo C.R., Hyman B.T., Bacskai B.J. (2009). Synchronous Hyperactivity and Intercellular Calcium Waves in Astrocytes in Alzheimer Mice. Science.

[38] Santello M., Toni N., Volterra A. (2019). Astrocyte function from information processing to cognition and cognitive impairment. Nat. Neurosci..

[39] Blanco-Suárez E., Caldwell A.L.M., Allen N.J. (2017). Role of astrocyte-synapse interactions in CNS disorders. J. Physiol..

[40] Bally B.P., Farmer W.T., Jones E.V., Jessa S., Kacerovsky J.B., Mayran A., Peng H., Lefebvre J.L., Drouin J., Hayer A. (2020). Human iPSC-derived Down syndrome astrocytes display genome-wide perturbations in gene expression, an altered adhesion profile, and increased cellular dynamics. Hum. Mol. Genet..

[41] Mizuno G.O., Wang Y., Shi G., Wang Y., Sun J., Papadopoulos S., Broussard G.J., Unger E.K., Deng W., Weick J. (2018). Aberrant Calcium Signaling in Astrocytes Inhibits Neuronal Excitability in a Human Down Syndrome Stem Cell Model. Cell Rep..

[42] Mito T., Becker L.E. (1993). Developmental Changes of S-100 Protein and Glial Fibrillary Acidic Protein in the Brain in Down Syndrome. Exp. Neurol..

[43] J∅Rgensen O.S., Brooksbank B.W., Balazs R. (1990). Neuronal plasticity and astrocytic reaction in Down syndrome and Alzheimer disease. J. Neurol. Sci..

[44] Quinlan R.A., Brenner M., Goldman J.E., Messing A. (2007). GFAP and its role in Alexander disease. Exp. Cell Res..

[45] Laurence J.A., Fatemi S.H. (2005). Glial fibrillary acidic protein is elevated in superior frontal, parietal and cerebellar cortices of autistic subjects. Cerebellum.

[46] Wu Z., Guo Z., Gearing M., Chen G. (2014). Tonic inhibition in dentate gyrus impairs long-term potentiation and memory in an Alzheimer’s disease model. Nat. Commun..

[47] Jo S., Yarishkin O., Hwang Y.J., Chun Y.E., Park M., Woo D.H., Bae J.Y., Kim T., Lee J., Chun H. (2014). GABA from reactive astrocytes impairs memory in mouse models of Alzheimer’s disease. Nat. Med..

[48] Gerlai R., Wojtowicz J.M., Marks A., Roder J. (1995). Overexpression of a calcium-binding protein, S100 beta, in astrocytes alters synaptic plasticity and impairs spatial learning in transgenic mice. Learn. Mem..

[49] Durkee C.A., Araque A. (2019). Diversity and Specificity of Astrocyte–neuron Communication. Neuroscience.

[50] Panatier A., Robitaille R. (2016). Astrocytic mGluR5 and the tripartite synapse. Neuroscience.

[51] Haydon P.G. (2001). Glia: Listening and talking to the synapse. Nat. Rev. Neurosci..

[52] Araque A., Carmignoto G., Haydon P.G., Oliet S.H.R., Robitaille R., Volterra A. (2014). Gliotransmitters Travel in Time and Space. Neuron.

[53] Ben Achour S., Pont-Lezica L., Béchade C., Pascual O. (2010). Is astrocyte calcium signaling relevant for synaptic plasticity?. Neuron Glia Biol..

[54] Mederos S., Perea G. (2019). GABAergic-astrocyte signaling: A refinement of inhibitory brain networks. Glia.

[55] Cavaccini A., Durkee C., Kofuji P., Tonini R., Araque A. (2020). Astrocyte Signaling Gates Long-Term Depression at Corticostriatal Synapses of the Direct Pathway. J. Neurosci..

[56] Araque A., Parpura V., Sanzgiri R.P., Haydon P.G. (1999). Tripartite synapses: Glia, the unacknowledged partner. Trends Neurosci..

[57] Volterra A., Liaudet N., Savtchouk I. (2014). Astrocyte Ca^2+^ signalling: An unexpected complexity. Nat. Rev. Neurosci..

[58] Savtchouk I., Volterra A. (2018). Gliotransmission: Beyond Black-and-White. J. Neurosci..

[59] Ventura R., Harris K.M. (1999). Three-Dimensional Relationships between Hippocampal Synapses and Astrocytes. J. Neurosci..

[60] Bushong E.A., Martone M.E., Jones Y.Z., Ellisman M.H. (2002). Protoplasmic Astrocytes in CA1 Stratum Radiatum Occupy Separate Anatomical Domains. J. Neurosci..

[61] Theodosis D.T. (2002). Oxytocin-Secreting Neurons: A Physiological Model of Morphological Neuronal and Glial Plasticity in the Adult Hypothalamus. Front. Neuroendocr..

[62] Nishida H., Okabe S. (2007). Direct Astrocytic Contacts Regulate Local Maturation of Dendritic Spines. J. Neurosci..

[63] Hirrlinger J., Hülsmann S., Kirchhoff F. (2004). Astroglial processes show spontaneous motility at active synaptic terminals in situ. Eur. J. Neurosci..

[64] Chung W.-S., Allen N.J., Eroglu C. (2015). Astrocytes Control Synapse Formation, Function, and Elimination. Cold Spring Harb. Perspect. Biol..

[65] Van Horn M.R., Ruthazer E.S. (2019). Glial regulation of synapse maturation and stabilization in the developing nervous system. Curr. Opin. Neurobiol..

[66] Christopherson K.S., Ullian E.M., Stokes C.C., Mullowney C.E., Hell J.W., Agah A., Lawler J., Mosher D.F., Bornstein P., Barres B.A. (2005). Thrombospondins are Astrocyte-Secreted Proteins that Promote CNS Synaptogenesis. Cell.

[67] Nishiyama H., Knöpfel T., Endo S., Itohara S. (2002). Glial protein S100B modulates long-term neuronal synaptic plasticity. Proc. Natl. Acad. Sci. USA.

[68] Morquette P., Verdier D., Kadala A., Féthière J., Philippe A.G., Robitaille R., Kolta A. (2015). An astrocyte-dependent mechanism for neuronal rhythmogenesis. Nat. Neurosci..

[69] Ahlemeyer B., Beier H., Semkova I., Schaper C., Krieglstein J. (2000). S-100β protects cultured neurons against glutamate- and staurosporine-induced damage and is involved in the antiapoptotic action of the 5 HT1A-receptor agonist, Bay x 3702. Brain Res..

[70] Mori T., Tan J., Arendash G.W., Koyama N., Nojima Y., Town T. (2008). Overexpression of Human S100B Exacerbates Brain Damage and Periinfarct Gliosis after Permanent Focal Ischemia. Stroke.

[71] Villarreal A., Avilés-Reyes R., Angelo M.F., Reines A.G., Ramos A.J. (2011). S100B alters neuronal survival and dendrite extension via RAGE?mediated NF??B signaling. J. Neurochem..

[72] Benchenane K., Tiesinga P.H., Battaglia F.P. (2011). Oscillations in the prefrontal cortex: A gateway to memory and attention. Curr. Opin. Neurobiol..

[73] Escuela D.O.B., Carlsson J., Ambrogini P., Narváez M., Wydra K., Tarakanov A.O., Li X., Millón C., Ferraro L., Cuppini R. (2017). Understanding the Role of GPCR Heteroreceptor Complexes in Modulating the Brain Networks in Health and Disease. Front. Cell. Neurosci..

[74] Durkee C.A., Covelo A., Lines J., Kofuji P., Aguilar J., Araque A. (2019). G i/o protein-coupled receptors inhibit neurons but activate astrocytes and stimulate gliotransmission. Glia.

[75] Doron A., Goshen I. (2018). Investigating the transition from recent to remote memory using advanced tools. Brain Res. Bull..

[76] Frankland P.W., Bontempi B. (2005). The organization of recent and remote memories. Nat. Rev. Neurosci..

[77] Moscovitch M., Cabeza R., Winocur G., Nadel L. (2016). Episodic Memory and Beyond: The Hippocampus and Neocortex in Transformation. Annu. Rev. Psychol..

[78] Araujo B.H.S., Kaid C., De Souza J.S., Da Silva S.G., Goulart E., Caires L.C.J., Musso C.M., Torres L.B., Ferrasa A., Herai R. (2017). Down Syndrome iPSC-Derived Astrocytes Impair Neuronal Synaptogenesis and the mTOR Pathway In Vitro. Mol. Neurobiol..

[79] Murphy G.M., Ellis W.G., Lee Y.-L., Stultz K.E., Shrivastava R., Tinklenberg J.R., Eng L.F. (1992). Chapter 40: Astrocytic Gliosis in the Amygdala in Down’s Syndrome and Alzheimer’s Disease.

[80] Guttenplan K.A., Stafford B.K., El-Danaf R.N., Adler D.I., Münch A.E., Weigel M.K., Huberman A.D., Liddelow S.A. (2020). Neurotoxic Reactive Astrocytes Drive Neuronal Death after Retinal Injury. Cell Rep..

[81] Kia A., McAvoy K., Krishnamurthy K., Trotti D., Pasinelli P. (2018). Astrocytes expressing ALS-linked mutant FUS induce motor neuron death through release of tumor necrosis factor-alpha. Glia.

[82] Sofroniew M. (2015). Astrogliosis. Cold Spring Harb. Perspect. Biol..

[83] Pekny M., Pekna M. (2014). Astrocyte Reactivity and Reactive Astrogliosis: Costs and Benefits. Physiol. Rev..

[84] Sofroniew M. (2009). Molecular dissection of reactive astrogliosis and glial scar formation. Trends Neurosci..

[85] Griffin W., Sheng J.G., McKenzie J.E., Royston M.C., Gentleman S.M., Brumback R.A., Cork L.C., Del Bigio M.R., Roberts G.W., Mrak R.E. (1999). Life-long Overexpression of S100β in Down’s Syndrome: Implications for Alzheimer Pathogenesis. Neurobiol. Aging.

[86] Banerjee A., Ifrim M.F., Valdez A.N., Raj N., Bassell G.J. (2018). Aberrant RNA translation in fragile X syndrome: From FMRP mechanisms to emerging therapeutic strategies. Brain Res..

[87] Dockendorff T.C., Labrador M. (2018). The Fragile X Protein and Genome Function. Mol. Neurobiol..

[88] Reiss A.L., Aylward E., Freund L.S., Joshi P.K., Bryan R.N. (1991). Neuroanatomy of fragile X syndrome: The posterior fossa. Ann. Neurol..

[89] Sabaratnam M. (2000). Pathological and neuropathological findings in two males with fragile-X syndrome. J. Intellect. Disabil. Res..

[90] (1994). The Dutch-Belgian Fragile X Consorthrum. Fmr1 knockout mice: A model to study fragile X mental retardation. Cell.

[91] Higashimori H., Morel L., Huth J., Lindemann L., Dulla C., Taylor A., Freeman M., Yang Y. (2013). Astroglial FMRP-dependent translational down-regulation of mGluR5 underlies glutamate transporter GLT1 dysregulation in the fragile X mouse. Hum. Mol. Genet..

[92] Bear M.F., Huber K.M., Warren S.T. (2004). The mGluR theory of fragile X mental retardation. Trends Neurosci..

[93] Dölen G., Osterweil E., Rao B.S.S., Smith G.B., Auerbach B.D., Chattarji S., Bear M.F. (2007). Correction of Fragile X Syndrome in Mice. Neuron.

[94] Veloz M.F.V., Buijsen R.A., Willemsen R., Cupido A., Bosman L.W., Koekkoek S.K.E., Potters J.W., Oostra B.A., De Zeeuw C.I. (2012). The effect of an mGluR5 inhibitor on procedural memory and avoidance discrimination impairments in Fmr1 KO mice. Genes Brain Behav..

[95] Pop A.S., Levenga J., De Esch C.E.F., Buijsen R.A., Nieuwenhuizen I.M., Li T., Isaacs A., Gasparini F., Oostra B.A., Willemsen R. (2012). (Rob) Rescue of dendritic spine phenotype in Fmr1 KO mice with the mGluR5 antagonist AFQ056/Mavoglurant. Psychopharmacology.

[96] Aloisi E., Le Corf K., Dupuis J., Zhang P., Ginger M., Labrousse V., Spatuzza M., Haberl M.G., Costa L., Shigemoto R. (2017). Altered surface mGluR5 dynamics provoke synaptic NMDAR dysfunction and cognitive defects in Fmr1 knockout mice. Nat. Commun..

[97] Carroll R.C., Lissin D.V., Von Zastrow M., Nicoll R.A., Malenka R.C. (1999). Rapid redistribution of glutamate receptors contributes to long-term depression in hippocampal cultures. Nat. Neurosci..

[98] Snyder E.M., Philpot B.D., Huber K.M., Dong X., Fallon J.R., Bear M.F. (2001). Internalization of ionotropic glutamate receptors in response to mGluR activation. Nat. Neurosci..

[99] Zakharenko S.S., Zablow L., Siegelbaum S.A. (2002). Altered presynaptic vesicle release and cycling during mGluR-dependent LTD. Neuron.

[100] Huber K.M., Gallagher S.M., Warren S.T., Bear M.F. (2002). Altered synaptic plasticity in a mouse model of fragile X mental retardation. Proc. Natl. Acad. Sci. USA.

[101] Yuskaitis C.J., Beurel E., Jope R.S. (2010). Evidence of reactive astrocytes but not peripheral immune system activation in a mouse model of Fragile X syndrome. Biochim. Biophys. Acta (BBA) Mol. Basis Dis..

[102] Pacey L.K.K., Guan S., Tharmalingam S., Thomsen C., Hampson D.R. (2015). Persistent astrocyte activation in the fragile X mouse cerebellum. Brain Behav..

[103] Cao Z., Hulsizer S., Cui Y., Pretto D.L., Kim K.H., Hagerman P.J., Tassone F., Pessah I.N. (2013). Enhanced Asynchronous Ca^2+^Oscillations Associated with Impaired Glutamate Transport in Cortical Astrocytes ExpressingFmr1Gene Premutation Expansion. J. Biol. Chem..

[104] Asch A.S., Leung L.L., Shapiro J., Nachman R.L. (1986). Human brain glial cells synthesize thrombospondin. Proc. Natl. Acad. Sci. USA.

[105] Yu K., Ge J., Summers J.B., Li F., Liu X., Ma P., Kaminski J., Zhuang J. (2008). TSP-1 Secreted by Bone Marrow Stromal Cells Contributes to Retinal Ganglion Cell Neurite Outgrowth and Survival. PLoS ONE.

[106] Adams J.C., Tucker R.P. (2000). The thrombospondin type 1 repeat (TSR) superfamily: Diverse proteins with related roles in neuronal development. Dev. Dyn..

[107] Lu Z., Kipnis J. (2010). Thrombospondin 1—A key astrocyte-derived neurogenic factor. FASEB J..

[108] Pinter J.D., Eliez S., Schmitt J.E., Capone G.T., Reiss A.L. (2001). Neuroanatomy of Down’s Syndrome: A High-Resolution MRI Study. Am. J. Psychiatry.

[109] Raz N., Torres I.J., Briggs S.D., Spencer W.D., Thornton A.E., Loken W.J., Gunning F.M., McQuain J.D., Driesen N.R., Acker J.D. (1995). Selective neuroanatornic abnormalities in Down’s syndrome and their cognitive correlates: Evidence from MRI morphometry. Neurology.

[110] De Lagran M.M., Benavides-Piccione R., Ballesteros-Yanez I., Calvo M., Morales M., Fillat C., DeFelipe J., Ramakers G.J.A., Dierssen M. (2012). Dyrk1A Influences Neuronal Morphogenesis Through Regulation of Cytoskeletal Dynamics in Mammalian Cortical Neurons. Cereb. Cortex.

[111] Lott I., Dierssen M. (2010). Cognitive deficits and associated neurological complications in individuals with Down’s syndrome. Lancet Neurol..

[112] Griffin W.S., Stanley L.C., Ling C., White L., MacLeod V., Perrot L.J., White C.L., Araoz C. (1989). Brain interleukin 1 and S-100 immunoreactivity are elevated in Down syndrome and Alzheimer disease. Proc. Natl. Acad. Sci. USA.

[113] Lockrow J.P., Fortress A.M., Granholm A.-C.E. (2012). Age-Related Neurodegeneration and Memory Loss in Down Syndrome. Curr. Gerontol. Geriatr. Res..

[114] Goodison K.L., Parhad I.M., White C.L., Sima A.A.F., Clark A.W. (1993). Neuronal and Glial Gene Expression in Neocortex of Downʼs Syndrome and Alzheimerʼs Disease. J. Neuropathol. Exp. Neurol..

[115] Kanaumi T., Milenkovic I., Adle-Biassette H., Aronica E., Kovacs G.G. (2013). Non-neuronal cell responses differ between normal and Down syndrome developing brains. Int. J. Dev. Neurosci..

[116] Guidi S., Bonasoni P., Ceccarelli C., Santini D., Gualtieri F., Ciani E., Bartesaghi R. (2007). RESEARCH ARTICLE: Neurogenesis Impairment and Increased Cell Death Reduce Total Neuron Number in the Hippocampal Region of Fetuses with Down Syndrome. Brain Pathol..

[117] Hibaoui Y., Grad I., Letourneau A., Sailani M.R., Dahoun S., Santoni F.A., Gimelli S., Guipponi M., Pelte M.F., Bena F.S. (2013). Modelling and rescuing neurodevelopmental defect of D own syndrome using induced pluripotent stem cells from monozygotic twins discordant for trisomy 21. EMBO Mol. Med..

[118] Kurabayashi N., Nguyen M.D., Sanada K. (2015). DYRK 1A overexpression enhances STAT activity and astrogliogenesis in a Down syndrome mouse model. EMBO Rep..

[119] Lorenzi H.A., Reeves R.H. (2006). Hippocampal hypocellularity in the Ts65Dn mouse originates early in development. Brain Res..

[120] Contestabile A., Fíla T., Cappellini A., Bartesaghi R., Ciani E. (2009). Widespread impairment of cell proliferation in the neonate Ts65Dn mouse, a model for Down syndrome. Cell Prolif..

[121] Anderson A.J., Stoltzner S., Lai F., Su J., Nixon R.A. (2000). Morphological and biochemical assessment of DNA damage and apoptosis in Down syndrome and Alzheimer disease, and effect of postmortem tissue archival on TUNEL. Neurobiol. Aging.

[122] Halassa M.M., Fellin T., Takano H., Dong J.-H., Haydon P.G. (2007). Synaptic Islands Defined by the Territory of a Single Astrocyte. J. Neurosci..

[123] Colombo J.A., Reisin H.D., Jones M., Bentham C. (2005). Development of interlaminar astroglial processes in the cerebral cortex of control and Down’s syndrome human cases. Exp. Neurol..

[124] Zhang J.-M., Wang H.-K., Ye C.-Q., Ge W., Chen Y., Jiang Z.-L., Wu C.-P., Poo M.-M., Duan S. (2003). ATP Released by Astrocytes Mediates Glutamatergic Activity-Dependent Heterosynaptic Suppression. Neuron.

[125] Pascual O., Casper K.B., Kubera C., Zhang J., Revilla-Sanchez R., Sul J.-Y., Takano H., Moss S.J., McCarthy K., Haydon P.G. (2005). Astrocytic Purinergic Signaling Coordinates Synaptic Networks. Science.

[126] Dunwiddie T.V., Masino S.A. (2001). The Role and Regulation of Adenosine in the Central Nervous System. Annu. Rev. Neurosci..

[127] Lindquist B.E., Shuttleworth C.W. (2012). Adenosine receptor activation is responsible for prolonged depression of synaptic transmission after spreading depolarization in brain slices. Neuroscience.

[128] Risser D., Lubec G., Cairns N., Herrera-Marschitz M. (1997). Excitatory amino acids and monoamines in parahippocampal gyrus and frontal cortical pole of adults with down syndrome. Life Sci..

[129] Reynolds G.P., Warner C.E. (1988). Amino acid neurotransmitter deficits in adult Down’s syndrome brain tissue. Neurosci. Lett..

[130] Begni B., Brighina L., Fumagalli L., Andreoni S., Castelli E., Francesconi C., Del Bo R., Bresolin N., Ferrarese C. (2003). Altered glutamate uptake in peripheral tissues from Down Syndrome patients. Neurosci. Lett..

[131] Chen C., Jiang P., Xue H., Peterson S.E., Tran H.T., McCann A.E., Parast M.M., Li S., Pleasure D.E., Laurent L.C. (2014). Role of astroglia in Down’s syndrome revealed by patient-derived human-induced pluripotent stem cells. Nat. Commun..

[132] Belichenko P.V., Kleschevnikov A.M., Masliah E., Wu C., Takimoto-Kimura R., Salehi A., Mobley W.C. (2009). Excitatory-inhibitory relationship in the fascia dentata in the Ts65Dn mouse model of down syndrome. J. Comp. Neurol..

[133] Harashima C., Jacobowitz D.M., Stoffel M., Chakrabarti L., Haydar T.F., Siarey R.J., Galdzicki Z. (2006). Elevated Expression of the G-Protein-Activated Inwardly Rectifying Potassium Channel 2 (GIRK2) in Cerebellar Unipolar Brush Cells of a Down Syndrome Mouse Model. Cell. Mol. Neurobiol..

[134] Best T.K., Cramer N.P., Chakrabarti L., Haydar T.F., Galdzicki Z. (2011). Dysfunctional hippocampal inhibition in the Ts65Dn mouse model of Down syndrome. Exp. Neurol..

[135] Costa A.C., Grybko M.J. (2005). Deficits in hippocampal CA1 LTP induced by TBS but not HFS in the Ts65Dn mouse: A model of Down syndrome. Neurosci. Lett..

[136] Kurt M., Davies D.C., Kidd M., Dierssen M., Flórez J. (2000). Synaptic deficit in the temporal cortex of partial trisomy 16 (Ts65Dn) mice. Brain Res..

[137] Oka A., Takashima S. (1999). The up-regulation of metabotropic glutamate receptor 5 (mGluR5) in Down’s syndrome brains. Acta Neuropathol..

[138] Gibson J.R., Bartley A.F., Hays S.A., Huber K.M. (2008). Imbalance of Neocortical Excitation and Inhibition and Altered UP States Reflect Network Hyperexcitability in the Mouse Model of Fragile X Syndrome. J. Neurophysiol..

[139] Olmos-Serrano J.L., Paluszkiewicz S.M., Martin B.S., Kaufmann W.E., Corbin J.G., Huntsman M.M. (2010). Defective GABAergic Neurotransmission and Pharmacological Rescue of Neuronal Hyperexcitability in the Amygdala in a Mouse Model of Fragile X Syndrome. J. Neurosci..

[140] Ethridge L.E., White S.P., Mosconi M.W., Wang J., Byerly M.J., Sweeney J.A. (2016). Reduced habituation of auditory evoked potentials indicate cortical hyper-excitability in Fragile X Syndrome. Transl. Psychiatry.

[141] Iyer A.M., Van Scheppingen J., Milenkovic I., Anink J.J., Lim D., Genazzani A.A., Adle-Biassette H., Kovacs G.G., Aronica E. (2014). Metabotropic Glutamate Receptor 5 in Down’s Syndrome Hippocampus During Development: Increased Expression in Astrocytes. Curr. Alzheimer Res..

[142] Piers T.M., Kim D.H., Kim B.C., Regan P., Whitcomb D.J., Cho K. (2012). Translational Concepts of mGluR5 in Synaptic Diseases of the Brain. Front. Pharm..

[143] Shiraishi-Yamaguchi Y., Furuichi T. (2007). The Homer family proteins. Genome Biol..

[144] Won H., Lee H.-R., Gee H.Y., Mah W., Kim J.-I., Lee J., Ha S., Chung C., Jung E.S., Cho Y.S. (2012). Autistic-like social behaviour in Shank2-mutant mice improved by restoring NMDA receptor function. Nat. Cell Biol..

[145] Gregory K.J., Dong E.N., Meiler J., Conn P.J. (2011). Allosteric modulation of metabotropic glutamate receptors: Structural insights and therapeutic potential. Neuropharmacology.

[146] Huber K.M. (2000). Role for Rapid Dendritic Protein Synthesis in Hippocampal mGluR-Dependent Long-Term Depression. Science.

[147] Lepannetier S., Gualdani R., Tempesta S., Schakman O., Seghers F., Kreis A., Yerna X., Slimi A., De Clippele M., Tajeddine N. (2018). Activation of TRPC1 Channel by Metabotropic Glutamate Receptor mGluR5 Modulates Synaptic Plasticity and Spatial Working Memory. Front. Cell. Neurosci..

[148] Sun Y., Lipton J.O., Boyle L.M., Madsen J.R., Goldenberg M.C., Pascual-Leone A., Sahin M., Rotenberg A. (2016). Direct current stimulation induces mGluR5-dependent neocortical plasticity. Ann. Neurol..

[149] Mor-Shaked H., Eiges R. (2018). Reevaluation of FMR1 Hypermethylation Timing in Fragile X Syndrome. Front. Mol. Neurosci..

[150] Liu X.S., Wu H., Krzisch M., Wu X., Graef J., Muffat J., Hnisz D., Li C.H., Yuan B., Xu C. (2018). Rescue of Fragile X Syndrome Neurons by DNA Methylation Editing of the FMR1 Gene. Cell.

[151] Lu J., McCarter M., Lian G., Esposito G., Capoccia E., Delli-Bovi L.C., Hecht J., Sheen V. (2016). Global hypermethylation in fetal cortex of Down syndrome due to DNMT3L overexpression. Hum. Mol. Genet..

[152] Jin S., Lee Y.K., Lim Y.C., Zheng Z., Lin X.M., Ng D.P.Y., Holbrook J.D., Law H.Y., Kwek K.Y.C., Yeo G.S.H. (2013). Global DNA Hypermethylation in Down Syndrome Placenta. PLoS Genet..

[153] Laufer B.I., Hwang H., Ciernia A.V., Mordaunt C.E., LaSalle J.M. (2019). Whole genome bisulfite sequencing of Down syndrome brain reveals regional DNA hypermethylation and novel disorder insights. Epigenetics.

[154] De Toma I., Ortega M., Catuara-Solarz S., Sierra C., Sabidó E., Dierssen M. (2020). Re-establishment of the epigenetic state and rescue of kinome deregulation in Ts65Dn mice upon treatment with green tea extract and environmental enrichment. Sci. Rep..

